# Nano-structuring, surface and bulk modification with a focused helium ion beam

**DOI:** 10.3762/bjnano.3.67

**Published:** 2012-08-08

**Authors:** Daniel Fox, Yanhui Chen, Colm C Faulkner, Hongzhou Zhang

**Affiliations:** 1 School of Physics and CRANN, Trinity College Dublin, Dublin 2, Republic of Ireland; 2 CRANN Advanced Microscopy Laboratory, Trinity College Dublin, Dublin 2, Republic of Ireland

**Keywords:** EELS, EFTEM, helium ion microscopy, nanofabrication, TEM

## Abstract

We investigate the ability of a focused helium ion beam to selectively modify and mill materials. The sub nanometer probe size of the helium ion microscope used provides lateral control not previously available for helium ion irradiation experiments. At high incidence angles the helium ions were found to remove surface material from a silicon lamella leaving the subsurface structure intact for further analysis. Surface roughness and contaminants were both reduced by the irradiation process. Fabrication is also realized with a high level of patterning acuity. Implantation of helium beneath the surface of the sample is visualized in cross section allowing direct observation of the extended effects of high dose irradiation. The effect of the irradiation on the crystal structure of the material is presented. Applications of the sample modification process are presented and further prospects discussed.

## Introduction

Ion beams are widely used to modify the physical and chemical properties of the surface of materials with a high degree of control. Ion beam irradiation can be used to modify and control a material’s optical [1], electrical [2], magnetic [3] and mechanical [4] properties. The gallium focused ion beam (FIB) microscope has been commercially available for twenty years. FIB microscopes have proven themselves as versatile tools with applications in a range of fields including biology [5], geology [6], materials science [7–8] and the semiconductor industry [9]. While the FIB has been adopted for many uses it is not without its limitations. The FIB uses gallium ions, a metallic element which is often considered a contaminant. The large momentum of the gallium ions in the FIB can have a very destructive effect on materials, greatly altering their crystal structure. The resolution of the FIB is limited by the energy spread of the gallium ions generated from the liquid metal ion source (LMIS). The sputter yield is also too large for acute patterning control over very short lateral distances. The recently developed Carl Zeiss Orion Plus helium ion microscope (HIM) is a new type of focused ion beam microscope. The HIM uses helium ions instead of gallium ions. Helium ions have a lower mass and therefore are less destructive than gallium ions. Helium ions are effectively non-contaminating. The source is a gas field ion source which does not suffer the energy spread and subsequent chromatic aberration which limits the resolution of the FIB [10]. Our HIM is capable of sub-nm resolution imaging with its <0.75 nm probe size. This makes it ideally suited to both high resolution imaging and also modification of materials with a higher level of control and precision than can be offered by other ion beam tools. The HIM has the unique ability to directly mill arbitrary patterns with sub 10 nm feature sizes. To date most of this work has been done on graphene [11–13]. The HIM can also directly write sub 10 nm features via precursor gas decomposition [14–15]. The depth of implantation of helium ions in a material can be varied by adjusting the beam energy [16]. Subsurface voids can be produced in such a fashion. Further exposure and subsequent growth of the void can be used to separate a thin film of material from a substrate [17], or to modify the optical properties of the surface [18]. Some other applications to date include imaging of chemical variations at high resolution [19], quantitative dopant contrast mapping [20] and imaging of uncoated biological materials [21]. In this work we further investigate the ability of the HIM to modify a material’s surface and structure using advanced transmission electron microscopy (TEM) techniques such as energy filtered TEM (EFTEM) and electron energy loss spectroscopy (EELS). We also present the limitations of this surface modification technique.

## Results and Discussion

**Figure 1:**
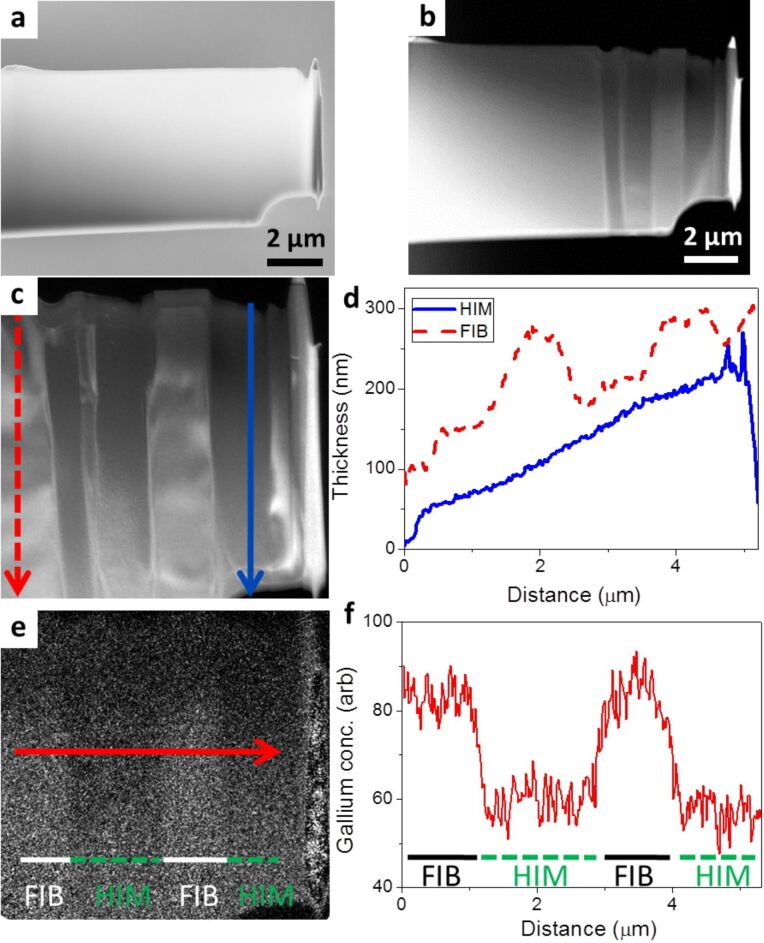
(a) SEM image of the silicon lamella (sample 1) after FIB preparation. (b) HAADF TEM image of the sample after three separate areas (observed as three vertical dark streaks) were modified by helium ions. (c) Thickness map of the modified area. The dashed red arrow is on an unmodified area of silicon. The solid blue arrow is on a HIM modified area. (d) The intensity profiles of the arrows from (c) are plotted, with the thickness calibrated. (e) EFTEM map indicating the distribution and concentration of gallium. The solid red line indicates the area from which (f) is plotted. The areas above the solid lines marked FIB have not been modified with helium ions, the areas above the dashed line marked HIM have been modified with helium ions. The integrated intensity profile from the arrow in (e) is plotted with the ion beam used to modify the region indicated below.

Sample 1 is a silicon lamella shown after FIB lift-out and thinning in the SEM image in Figure 1a. Figure 1b shows the TEM high angle annular dark field (HAADF) image of the lamella after HIM modification. The three dark vertical grooves indicate the areas modified in the HIM. In Figure 1b one effect of the helium ion modification process is clear; material is selectively removed from the sample sidewalls. The thickness map of the modified area is shown in Figure 1c. From this map we can calculate quantitative thickness values based on the mean free path of a 300 keV electron in silicon (180 nm) [22]. The arrows on the image indicate the regions from which the integrated intensity profiles were plotted in Figure 1d. The dashed red line is from a region prepared by gallium ions only. The solid blue line is from the helium ion modified area. The profiles both show an increase in thickness with increasing distance from the top of the lamella, indicating that the sample has a wedge shape. This increase is more gradual and noticeably smoother after helium ion modification. The modified area was observed to be consistently thinner than the unmodified region when compared at the same distance from the top of the lamella. The suitable area for TEM extends further from the top of the sample in the modified region, this results in a larger area useable for TEM in samples modified by the HIM. Figure 1e shows the EFTEM gallium map of the region. The areas of higher intensity in this map have a greater concentration of gallium contamination. The solid red arrow indicates the region from which the integrated intensity profile in Figure 1f was plotted. The solid white line is below the FIB prepared areas, while the dashed green line is beneath the areas further modified by helium ions. In Figure 1f the intensity profile from Figure 1e is plotted with the corresponding ion beam used to modify the area indicated below. This graph clearly shows a significant reduction in gallium contamination implanted by the FIB lift-out process. Typically around a 32% reduction in gallium concentration is achieved by HIM modification.

**Figure 2:**
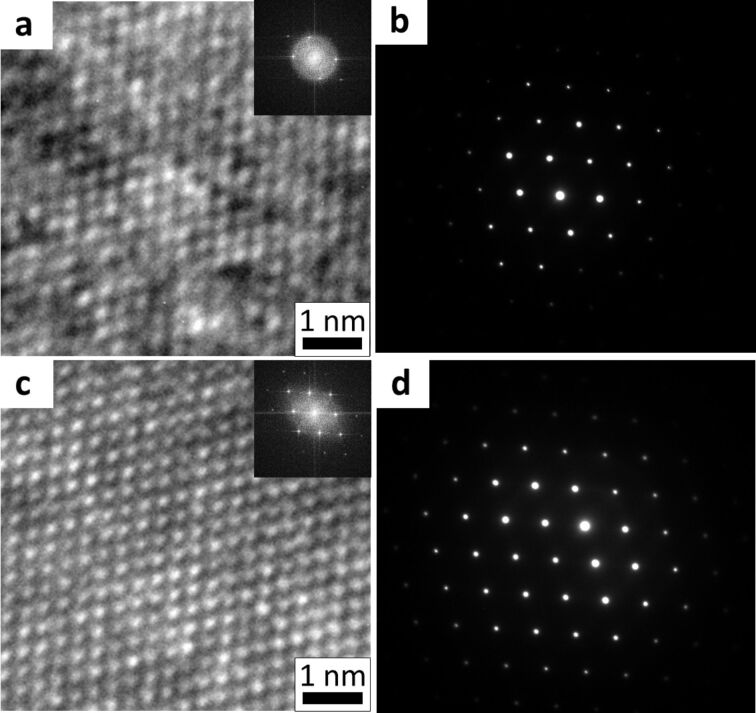
HRTEM (a) and diffraction (b) information from gallium finished region. HRTEM (c) and diffraction information (d) from helium modified region.

High resolution TEM (HRTEM) was performed on the HIM modified grooves and the unmodified sidewalls to afford further insight into the material modification. Figure 2a is the HRTEM image of the unmodified region of silicon; Figure 2b is the corresponding selected area diffraction (SAED) pattern from the region. Figure 2c is the HRTEM image of the HIM modified region of the sample, Figure 2d is the corresponding SAED pattern. Figure 2a displays a noisy HRTEM image when compared with that of Figure 2c from the HIM modified region of the sample. The inset FFT of the images also show the increase in high frequency information attained from the modified region. The uniform background contrast of the modified area indicates that it has a more uniform thickness. These images indicate that the amorphous layer of material on the sample, which contributes to background noise only, is reduced by HIM modification. Similarly, the SAED pattern in Figure 2b shows less information than that in Figure 2d. The extended high frequency information in the diffraction pattern recorded from the HIM modified region in Figure 2d indicates that this area of the sample is thinner, while still retaining its high quality crystalline structure. The diffraction patterns show that the sample was measured along the [110] direction.

**Figure 3:**
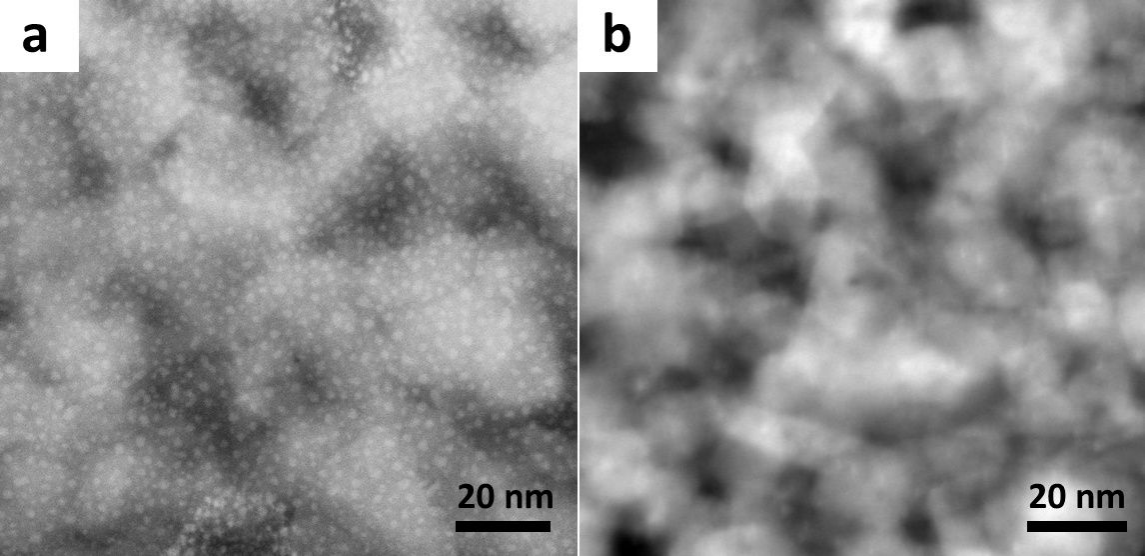
(a) HAADF image of the TiO_2_ sample (sample 2) after FIB lift-out. (b) HAADF image of the sample after HIM modification.

This analysis of sample 1 clearly shows us that HIM modification of a FIB prepared TEM lamella can be used to further reduce sample thickness while removing contamination and also retaining the crystallinity of the material. In order to investigate this polishing effect further we used the HIM to modify a TiO_2_ TEM lamella prepared by FIB lift-out. We called this sample 2. Figure 3a is a HAADF image of the sample after FIB lift-out, gallium contamination is observed as the small white spots on the image, the background noise is also large. The same sample was analyzed again after modification with 35 keV helium ions. The HAADF image of the modified region in Figure 3b shows a striking improvement as the surface agglomerations were removed and the contrast in the image was greatly improved.

**Figure 4:**
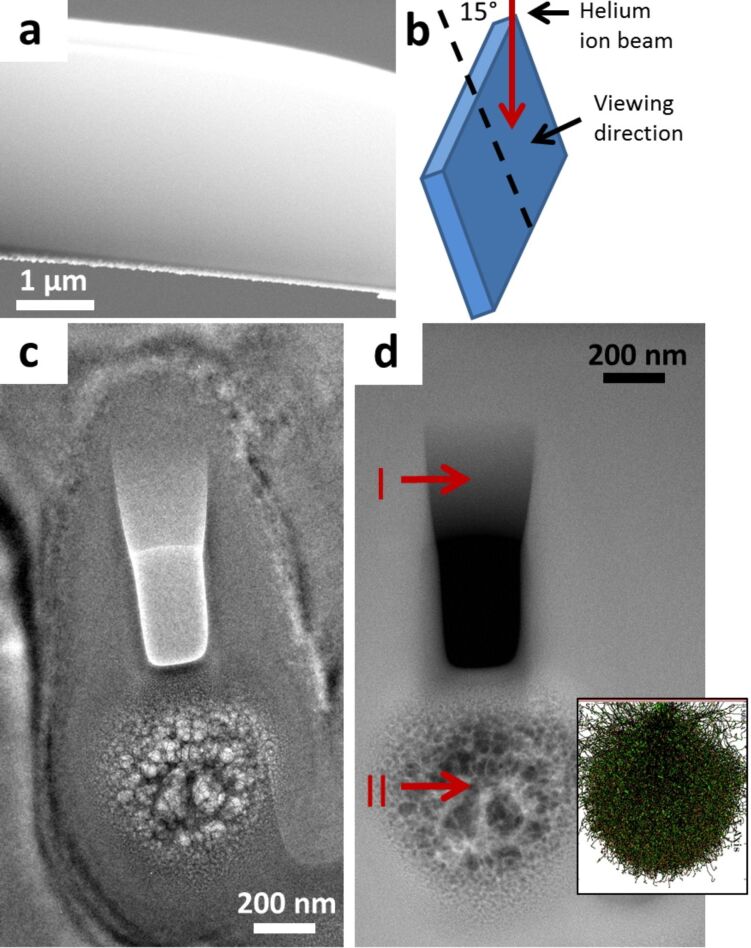
(a) SEM image of the silicon lamella (sample 3) after FIB lift-out and a 5 keV gallium ion polish. (b) Illustration of the geometry of the helium ion beam irradiation. The red arrow represents the helium ions which are incident on the face of the sample at an angle of 15°. (c) Bright field TEM image of the area modified by helium ions. (d) HAADF image of the modified area. “I” shows the location of the wedge shape and “II” shows the circular area with bubbles. Inset is a SRIM simulation of 35 keV helium ions in silicon with the same scale as the image.

**Figure 5:**
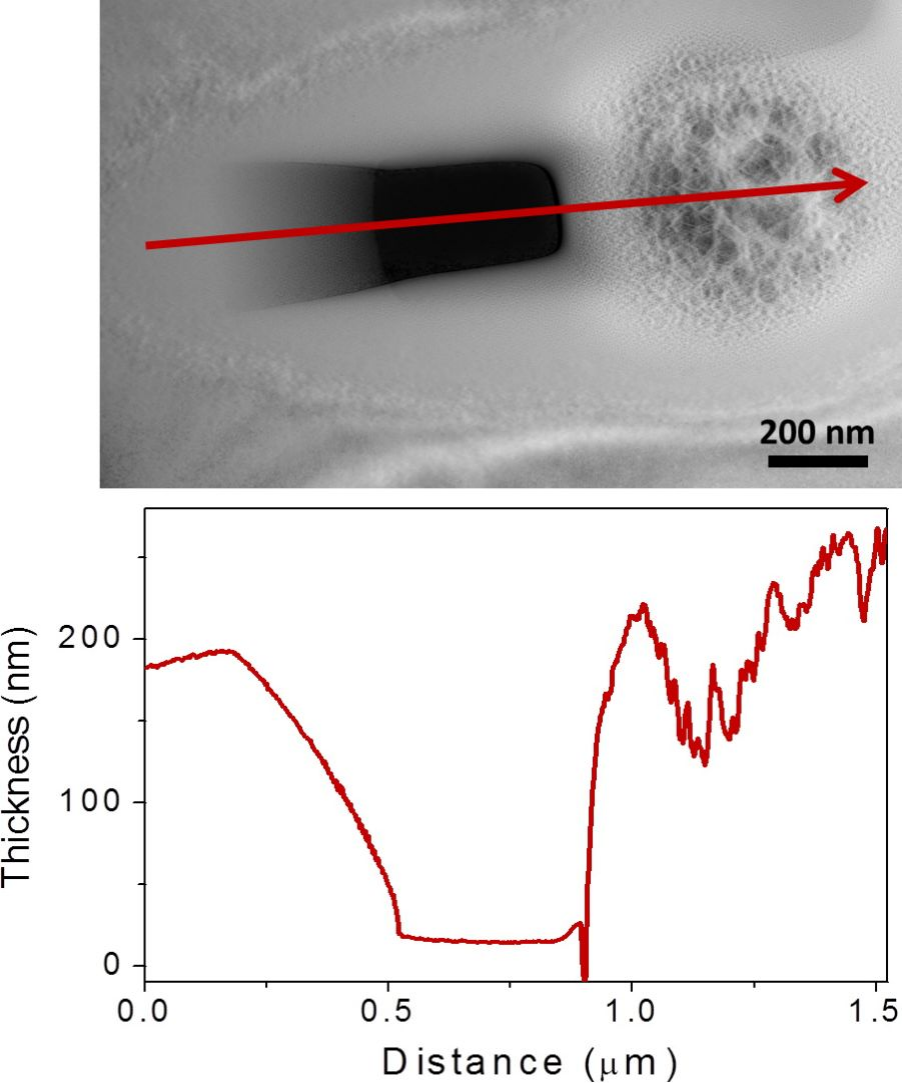
Thickness map of the modified region. The arrow indicates the area along which the integrated intensity profile below the image was plotted.

In samples 1 and 2 we have used lamellae finished only with 30 keV gallium ions in the FIB. A lower energy gallium ion beam can be used to produce lamellas with significantly less FIB induced damage [23]. FIBs with low energy capability have become more widely available over the past few years. We prepared the silicon lamella in sample 3 with a 5 keV gallium ion beam final polish in order to reduce FIB induced artifacts which would obscure our analysis of the patterning and subsurface modification effects of the HIM modification. Sample 3 is shown after FIB lift-out in the SEM image in Figure 4a. Figure 4b is an illustration of the beam–sample geometry used to modify the sample in the HIM. The sample sidewall was inclined 15° to the beam. This geometry was used in order to mill a wedge shape within the lamella in order to observe the minimum thickness dimensions which can be fabricated by HIM. This geometry also allows us to observe the extended effects of the modification process. Figure 4c is a bright field TEM image of the area of the sample after controlled sidewall modification by helium ion irradiation. Figure 4d is a HAADF image of the same area. A rectangular hole is observed at the center of the image, this was fabricated due to the high dose used. Below this hole is a circular region with rapid variations in contrast. This circular area has been heavily modified by the HIM. Figure 5 is a thickness map of the area. Below this map is the integrated intensity profile of the area indicated by the solid red arrow on the thickness map. This profile shows the sloping thickness of the wedge fabricated by helium ion irradiation, followed by the hole where the beam penetrated the lamella. Finally, the circular feature is observed to have rapidly varying thickness. The hole has a non-zero thickness due to the limitations of the thickness mapping process, such as its tendency to overestimate the thickness of very thin areas [24]. It is well known that helium ion irradiation, with an appropriate beam energy, can produce helium bubbles beneath the surface of a sample [25]. In this case the center of the circular feature is approximately at the implantation depth of 35 keV helium ions in silicon, about 318 nm (SRIM) [26]. This is made clear by the SRIM simulation image inset in Figure 4d showing the distribution of 35 keV helium ions in silicon; this simulation has the same scale as the image and correlates well with our experimental data. What we observe in this region is the implantation of helium ions, where the incident helium ions have been scattered by the silicon and have come to rest within the sample. These implanted ions then lead to the formation of bubbles beneath the surface which stretch and distort the silicon. The contrast observed corresponds to regions where helium bubbles have formed and silicon has been displaced.

**Figure 6:**
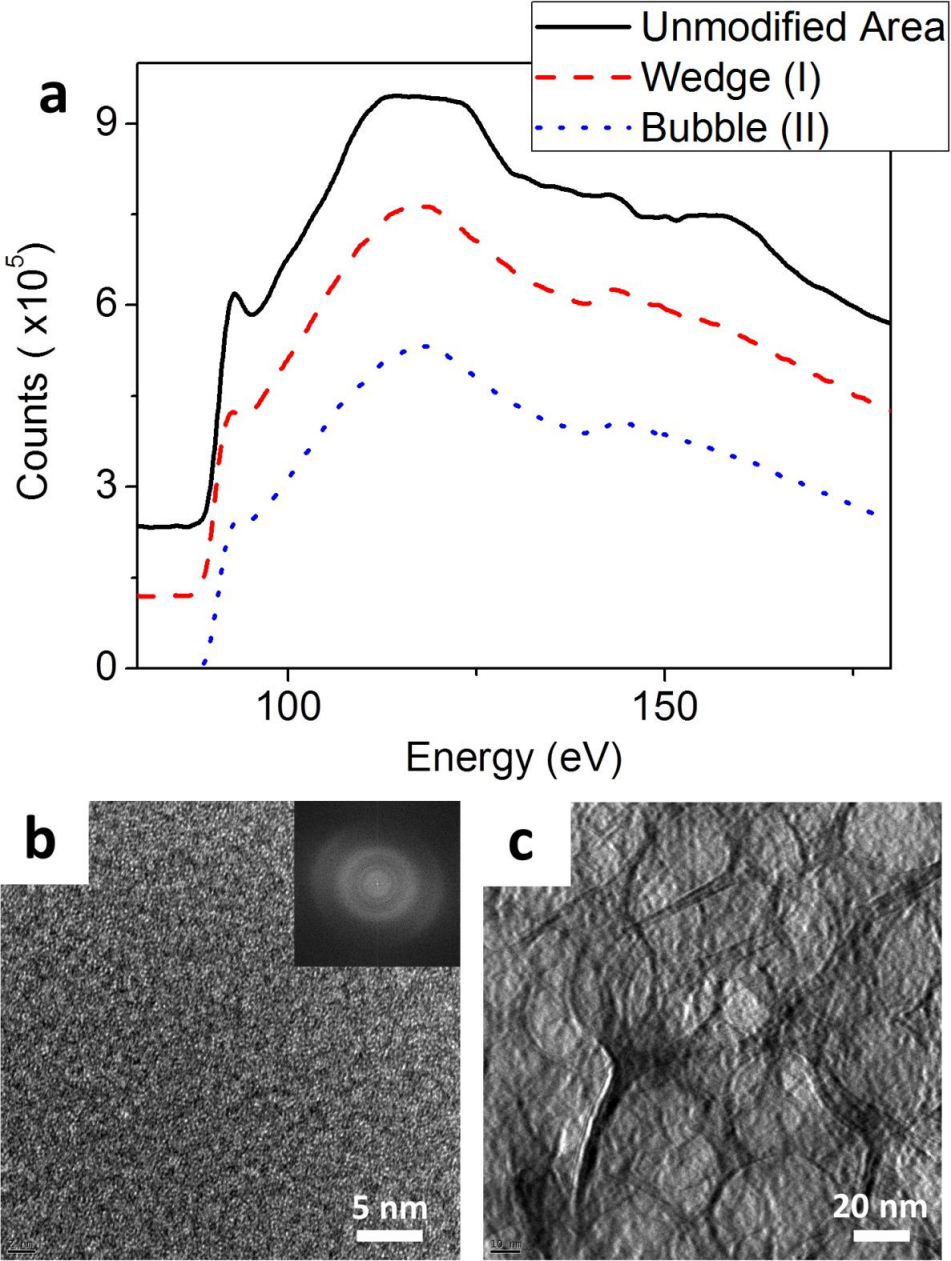
(a) EELS spectra of an unmodified area of silicon (solid black line), the HIM fabricated wedge marked I in Figure 4(d) (dashed red line) and the circular featured marked II in Figure 4(d) (dotted blue line). The spectra are offset for clarity. (b) HRTEM image of the wedge shaped area (I in Figure 4(d)) and FFT inset. (c) HRTEM of the circular feature (II in Figure 4(d)).

At this point we have described the morphological changes induced by a high dose of HIM irradiation on sample 3. We then investigated the effect of the HIM modification on the structure of the silicon. Figure 6a is a graph of the EELS spectra recorded from three different locations on the sample. The black solid line at the top was recorded at a region which was not modified by helium ion irradiation. The red dashed line in the middle was recorded from the wedge shape region fabricated by helium ion irradiation (marked “I” in Figure 4d). And finally the blue dotted line at the bottom was recorded at the circular feature (marked “II” in Figure 4d). When we analyzed our three EELS spectra in Figure 6a (spectra are offset for clarity) we found that the intensity of the first peak in the spectra at ≈99 eV was observed to degrade from the top spectrum to the bottom. We compared our data to the spectra for crystalline and amorphous silicon from an online database [27]. The intensity of the peak at ≈99 eV is used as an indication of the crystallinity of the silicon. A higher intensity indicates more crystallinity in that area, a lower intensity corresponds to an area which is more amorphous [28]. The top spectrum in our data corresponds to an area of high crystallinity, as indicated by the presence of a peak in this region of the spectrum. This result was to be expected as the spectrum was recorded from an unmodified region of the sample. However, the spectrum from the wedge shape area fabricated by the HIM (marked “I” in Figure 4d) shows a high degree of amorphization as the intensity of the first peak at ≈99 eV is greatly reduced. The spectrum from the area of the sample containing the bubbles (marked “II” in Figure 4d) shows an area which is even more amorphous again.

Figure 6b is a HRTEM image from the wedge area (marked “I” in Figure 4d) with an inset FFT of the image. This image shows only amorphous material is present at this location. Figure 6c is a HRTEM image of the area with the circular feature (marked “II” in Figure 4d). This image shows the presence of circles created by the growth of helium bubbles. No crystal structure was observed in this region either. We have observed that a high dose of HIM irradiation on sample 3 was used to fabricate a smooth wedge of material on a TEM lamella with minimum thickness dimensions of just a few tens of nanometers. This thickness may even be less than our thickness map indicates as significant errors may be present in the mapping process for very thin samples due to surface excitations, which can lead to overestimation of the thickness in this region [24]. The lateral dimensions of the pattern can also be tailored with a high degree of precision; the HIM can fabricate 4 nm nanopores quickly and reliably [29]. The EELS spectra and HRTEM from the HIM modified areas show that the wedge fabrication process caused significant amorphization of the sample in that region. The beam particles were also observed to implant in the sample below the wedge causing bubbles to form in the material, again resulting in significant amorphization of the silicon, as observed by EELS and HRTEM.

## Conclusion

We have demonstrated that a focused helium ion beam can modify a surface’s physical properties, such as crystallinity, roughness and thickness, in a controlled manner. 35 keV helium ions were used to produce a surface which was smoother than could be achieved by 30 keV FIB. Low energy FIB polishing can also improve the lamella quality; however the HIM polishing step has many benefits over the relatively broad beam of low energy FIB due to the its small probe size and the ion species used. The helium ion beam is non-contaminating and can even be used to selectively remove surface contaminants, such as the gallium contamination removal demonstrated here. Thickness dimensions can also be reduced to just a few tens of nanometers. This is critical for techniques in aberration corrected TEM such as atomically resolved EELS [30]. The higher imaging resolution of the HIM than the conventional SEM beam used in dual-beam FIB systems means that smaller features of interest can be located during sample preparation, prior to TEM. Clearly one application of this technique is as a highly useful step in the production of high quality TEM lamellae of silicon based devices, as well as a broader range of materials as demonstrated by our dramatic TiO_2_ sample quality improvement. Beyond TEM sample modification, it has been shown that a finely focused beam of helium ions can sputter material from a sample with a high level of control, allowing sub 10 nm features to be patterned. This high dose irradiation can also be used to modify the structure of a material, as demonstrated by our EELS and HRTEM results. The new models of the HIM feature variable acceleration voltage operation, our microscope is in the process of receiving this upgrade and further work will need to be done to assess the effect of reduced beam energies on sample modification. Helium ion irradiation is a widely studied field in nuclear physics. Helium ion irradiation has been used to modify mechanical [31], optical [25] and magnetic [32] properties of surfaces. The highly focused probe of the helium ion microscope provides a greater level of spatial control than previously available for such experiments.

## Experimental

An intrinsic silicon substrate was used as a base material for our experiments. The silicon was mounted on a lift-out sample holder and inserted into a Carl Zeiss Auriga CrossBeam FIB-SEM. A 12 µm long, 0.6 µm wide and 6 µm deep section of silicon was removed from the sample and transferred to an Omniprobe TEM lift-out grid by the in situ lift-out technique [33]. 30keV gallium ions were used to thin the silicon lamella to approximately 200 nm. The final thinning, to ≈100 nm, was done with a 20 pA beam of 30 keV gallium ions until the sample became almost transparent to a 5 keV electron beam, this is an indication that the sample is of sufficient thinness for TEM analysis. This is a common technique for TEM sample preparation. This process was then repeated to produce a second sample. Sample 2 is composed of TiO_2_ and was subjected to the same process as sample 1. Sample 3 is a silicon sample; it was treated with an extra final step. The sample face was tilted 2° into the beam and scanned with reduced energy gallium beam of 5 keV for one minute on each side, the beam current was 20 pA. A short dwell time and a large number of scan repeats were used for this step. This step is known to produce high quality TEM lamellae with a FIB induced amorphization and implant layer as thin as ≈2 nm, roughly ten times less than the damage layer produced by 30 keV gallium ions [23]. Sample 1 was then inserted into our HIM. The HIM beam energy was fixed at approximately 35 keV for all experiments. The lamella was loaded with the normal of the sidewall surface perpendicular to the ion beam, the sample was then tilted 1° into the beam. This geometry has been shown to produce surfaces with minimal damage and implantation layer thickness and reduced surface roughness in the FIB. A focused beam of helium ions was then scanned over a 500 nm wide region of the lamella sidewall. This step was conducted a number of times in adjacent regions in order to optimize the dose. The area used in our analysis was exposed to a dose of 3.4 × 10^16^ ions/cm^2^. The beam current used was 1.2 pA. The beam was rastered in a single scan over the area with a pixel spacing of 1 nm and a dwell time at each point of 1.3 × 10^−3^ s. The sample was rotated through 180° and the process was repeated on the opposite sidewall. The scanning time per sidewall was 68 s. This section of the sample was observed to have a reduced thickness after modification with helium ions. To investigate controlled modification for different materials sample 2 was treated with the same process. Sample 3 was then loaded into the HIM in the same upright manner. This sample was tilted 15° to the beam and a 300 × 200 nm area was exposed to a dose of 6.2 × 10^18^ ions/cm^2^. The beam current used was 3.4 pA. The beam was rastered in a single scan over the area with a pixel spacing of 0.7 nm and a dwell time at each point of 5 × 10^−3^ s. The total scanning time was 612 s. This process produced a hole straight through the lamella. The purpose of this exposure geometry was to produce a wedge shape of silicon within the lamella so that we could observe the minimum thickness dimensions which can be fabricated by HIM. It also allows us to analyze the subsurface modification effects due to helium ion implantation. All helium ion patterning was performed with the integrated pattern generator on the tool. For detailed analysis of the effects of our HIM modification these samples were analyzed in an FEI Titan 80-300 (S)TEM operating at 300 kV. High angle annular dark field (HAADF) images were recorded. HAADF images contain contrast due to sample thickness and composition. Thickness maps of samples 1 and 3 were recorded, these maps use energy filtering of electrons to provide quantitative thickness information. Energy filtered TEM (EFTEM) mapping of the gallium concentration and distribution in sample 1 was recorded. EELS spectra were recorded from sample 3. High resolution TEM (HRTEM) images and selected area electron diffraction (SAED) patterns were also recorded.

## Supporting Information

A full size HRTEM image of an unmodified region of sample 1 is available in Figure S1. A JEMS[34] software simulation of the HRTEM images is available in Figure S2. The simulated images illustrate the effect of crystal thickness on image contrast.

File 1 High resolution TEM imaging and simulation.
