# Supplementary material for: Nano-structuring, surface and bulk modification with a focused helium ion beam
Source: Beilstein J Nanotechnol. 2012 Aug 8;3:579–85. doi: 10.3762/bjnano.3.67 (PMC3458604; doi:10.3762/bjnano.3.67)
Supplement: File 1 — High resolution TEM imaging and simulation. [file Beilstein_J_Nanotechnol-03-579-s001.pdf]

Supporting Information

for

Nanostructuring, surface and bulk modification with a focused helium ion beam

Daniel Fox[1], Yanhui Chen[1], Colm C. Faulkner[2]and Hongzhou Zhang*[1]

Address: [1]School of Physics and CRANN, Trinity College Dublin, Dublin 2, Republic of Ireland and [2]CRANN Advanced Microscopy Laboratory, Trinity College Dublin, Dublin 2, Republic of Ireland

Email: Hongzhou.Zhang* - Hongzhou.Zhang@tcd.ie

*Corresponding author

High-resolution TEM imaging and simulation

In Figure 2a we found the background contrast to change throughout the image. A lower magnification image from the same region is shown in Figure S1. This contrast is absent in Figure 2c, the image is more uniform throughout. We have seen that Figure 2a comes from an area with a rough surface and non-uniform thickness in Figure 1d (dashed red line), when compared to the area of Figure 2c (solid blue line in Figure 1d). This background contrast can be attributed to the variation of thickness of the sample within the image. When we simulate the HRTEM images by viewing silicon along the [110] axis using the JEMS software package [1], we find that the contrast changes greatly with different thicknesses of the silicon crystal. The simulated images are shown in Figure S2. These images are simulated with all of the same parameters such as defocus (−78 nm), the only varying parameter being the crystal thickness, which gets 7.7 nm thinner in each image.

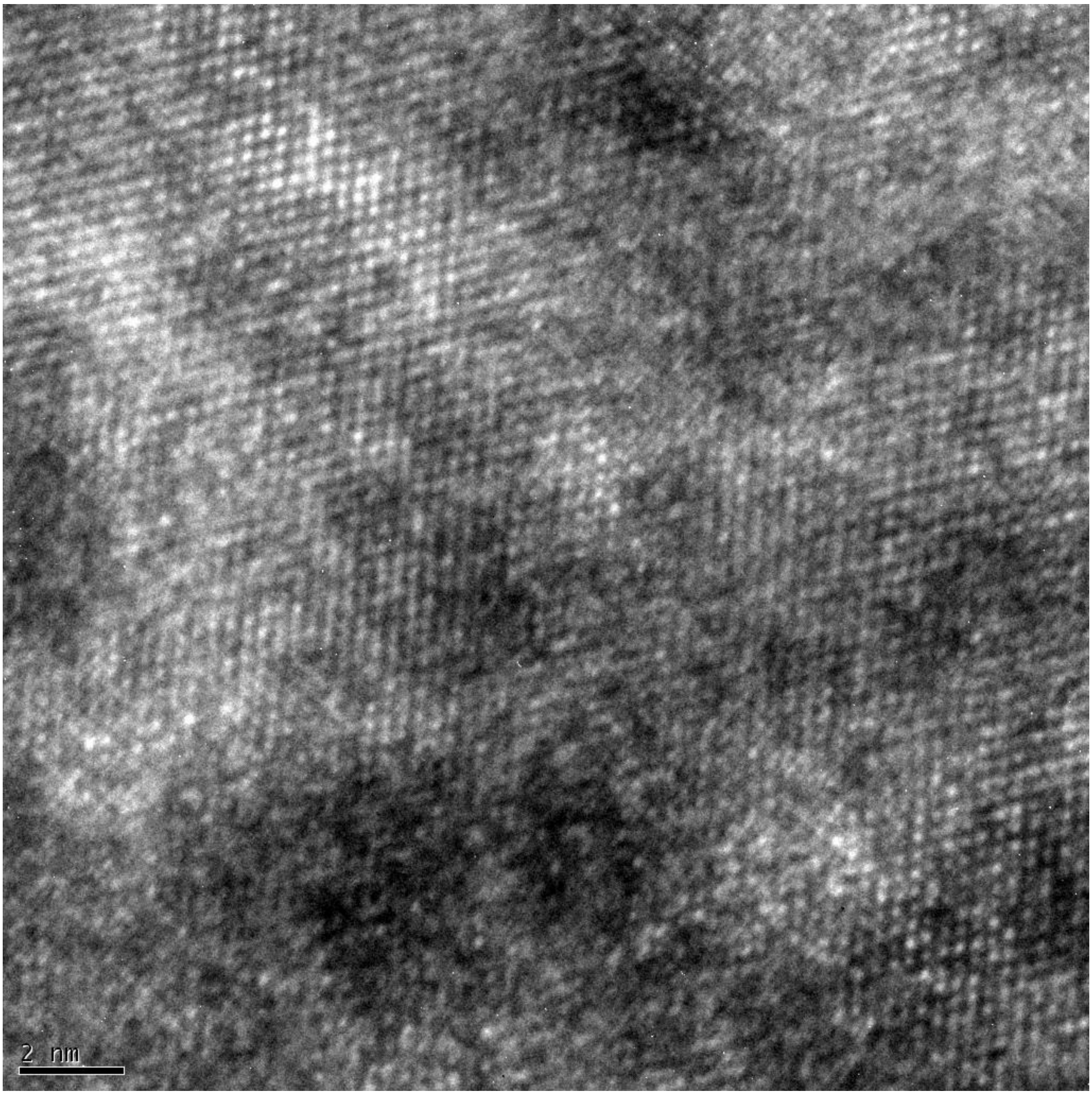

Figure S1: HRTEM image from an area of the silicon sample that has not been modified by helium ions.

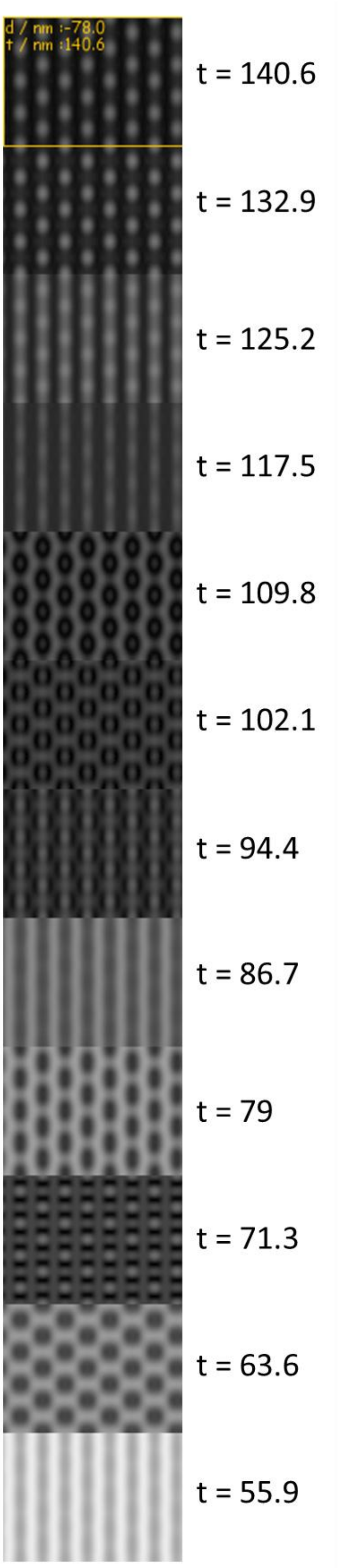

Figure S2: JEMS simulation of HRTEM images of silicon[110] with various different crystal thicknesses.

References

1. jems opening page sept 21 2004.

http://cimewww.epfl.ch/people/stadelmann/jemsWebSite/jems.html (accessed March 5, 2012).
